# Actual perspective on off‐pump transapical artificial chord implantation

**DOI:** 10.1111/jocs.16330

**Published:** 2022-02-19

**Authors:** Matteo Saccocci, Andrea Colli

**Affiliations:** ^1^ Unit of Cardiac Surgery—Cardiovascular Department Poliambulanza Foundation Hospital Brescia Italy; ^2^ Department of Surgical, Medical and Molecular Pathology and Critical Care Medicine University of Pisa Pisa Italy

**Keywords:** Harpoon, mitral regurgitation, mitral valve repair, Mitralstitch, MVR, Neochord, neochord implantation, transcatheter

## Abstract

Mitral valve repair (MVR) is undisputedly associated with better clinical and functional outcomes than any other type of valve substitute. Conventional mitral valve surgery in dedicated high‐volume centers can assure excellent results in terms of mortality and freedom from mitral regurgitation (MR) recurrence but requires cardiopulmonary bypass (CPB) and cardioplegic heart arrest. Trying to replicate the percentage of success of surgical MVR is the aim of all new transcatheter mitral dedicated devices. In particular, transapical beating‐heart mitral valve repair by artificial chordae implantation with transesophageal echocardiography guidance is an expanding field. The safety and feasibility of the procedure have already been largely demonstrated with Neochord and more recently with Harpoon systems. Wang et al. present the outcomes of the first‐in‐human experience using a novel artificial chordae implantation device, the Mitralstitch system. Despite a quite small cohort of only 10 patients treated, 1‐year results are satisfying and comparable with the early experience with former devices (4 patients with moderate or more MR recurrence). The comparison with surgical MVR is still unfavorable and requires further studies and significant procedure improvement. However, the device permits the treatment of anterior and posterior leaflets prolapse and performs quite easily edge‐to‐edge reparation. It will be interesting to evaluate longer follow‐up in larger cohorts of patients as well as the possibility to shift to the transfemoral approach.

Results of surgical reparation in mitral valve disease are outstanding, particularly for degenerative mitral regurgitation (DMR), where perioperative mortality tends to zero and freedom from reoperation is extremely high even at long‐term follow‐up. Nowadays, most high‐volume centers have solid and extensive minimally invasive surgery programs for the treatment of DMR patients.

Nevertheless, the constant desire to minimize the procedural impact on the patient has driven the development of various transcatheter solutions that could achieve a surgical‐like result without the need of a cardioplegic arrested heart and cardiopulmonary bypass (CPB). Unlike surgery, which can address and solve mitral insufficiency contemporarily treating all the components of mitral valve apparatus, the transcatheter devices, actually present in the market, work on leaflet or annulus separately. In their interesting paper, Wang et al. present the initial experience with the MitralStitch™ System (Hangzhou DeJin Medtech Co., Ltd.). This relatively new device,^1^ follows the footsteps of Neochord (Neochord, Inc.) and Harpoon (Edwards Lifesciences) and permits the positioning of polytetrafluoroethylene (ePTFE) artificial chordae through a transapical off‐pump ventricular access. The efficacy and safety of this solution have already been demonstrated mainly thanks to the worldwide expansion of the Neochord experience.^2^, ^3^, ^4^, ^5^, ^6^, ^7^, ^8^, ^9^, ^10^, ^11^, ^12^, ^13^, ^14^, ^15^, ^16^, ^17^, ^18^, ^19^, ^20^, ^21^, ^22^, ^23^, ^24^, ^25^, ^26^

The market launch of this new device, as the development of many others under preclinical investigation,^7^, ^27^, ^28^, ^29^, ^30^, ^31^, ^32^ demonstrates, if there was still a need, the importance of a novel and alternative approach in mitral valve disease treatment. Artificial chordal implantation is an already validated technique in mitral valve surgery with proven results in acute and long‐term outcomes,^33^, ^34^ usually associated with prosthetic ring annuloplasty. Trying to replicate surgery results, avoiding CPB and cardioplegic arrest, is ambitious, but it is worth the effort. Pursuing this aim, many studies have been published to optimize the Neochord procedure and the patients' selection,^7^, ^27^, ^28^, ^29^, ^30^, ^31^, ^32^ paving the road to better results for all the new devices entering the clinical arena. Therefore, the good short‐term outcomes reported by Wang et al. are not surprising and could potentially be confirmed at mid‐ and long‐term follow‐up.

The MitralStitch device presents some new features that could positively impact the procedure. The Nitinol frame of the retractable arm, enhancing the echocardiographic visibility of the device tip, is an interesting solution to facilitate the leaflet grasping maintaining a small profile of the device shaft. Unlike Harpoon, actually commercially approved only for posterior leaflet disease, this new product can be used to treat both leaflets as the Neochord system.^35^ The device has a small footprint allowing a theoretical relatively smooth transition to the transfemoral approach.

The durability of transapical artificial chordal can hardly be predicted at the moment. The results of an extensive large observational registry‐based in Germany on the Neochord system (NCT NCT04190602; The AcChord Study: A Multicenter Post‐Market Observational Registry of the NeoChord Artificial Chordae Delivery System) are expected between 2022 and 2027. Single cases of chordal rupture are reported for both Harpoon^16^ and Neochord^36^ systems but seem to be linked more to initial technical implantation errors at the beginning of the learning curve than to intrinsic chordal factors.^29^

As mentioned before, surgical valve reparation has proven to be safe and effective in the short‐, medium‐, and long term. However, direct comparison between surgery and transcatheter procedures is not always straight and easy, especially for mitral valve where solutions like MitraClip (Abbott Vascular) or Pascal (Edwards Lifesciences) are actually reserved for high‐risk patients. Neochord and Harpoon procedures have been used in both high‐ and low‐risk patients; overall results are encouraging but still distant from surgery. Citing Ilir Hysi and Olivier Fabre^37^ in their commentary to the 1‐year results of Harpoon published by Gammie et al.,^23^ “Where do we set the limit” for mitral valve repair? Published results of transapical chordal implantation show significant percentages of more than mild residual mitral regurgitation.^5^, ^6^, ^8^, ^11^, ^38^, ^39^ Gammie et al.^23^ introducing Harpoon results in their series of more than 60 patients show at 30 days 13% of moderate MR and 2% of severe MR. At 1 year, these percentages increase up to 23% and 2% for moderate and severe MR, respectively; 13% of the patients required reoperations. Lozos et al.,^10^ publishing the results of the first experience in Greece with Neochord DS1000, presented a 16% and 7% of moderate and severe residual MR, respectively. These results are usually considered acceptable and encouraging, but if compared with surgery, even if being able to avoid CPB and cardioplegic arrest, could sound not so brilliant. Even more, if we note that the enrolled patients are usually affected by single P2 prolapse according to the latest evidence on optimized patients’ selection.^7^, ^40^

The existence of a learning curve is a well‐known phenomenon and could be accountable for the results of these initial experiences. Colli et al.^41^ calculated that the team required a threshold of 40 Neochord interventions to optimize all the steps of the procedure. In fact, by analyzing the results and the mechanisms of recurrent regurgitation, the authors identified three major classes of causes: inappropriate patients selection, technical issue, and leaflet curling during the procedure.^42^ More than a paper has tried to directly compare the Neochord system and surgical mitral valve repair. The study by Zorinas et al.^43^ on a cohort of 169 patients treated for MR (78 Neochord procedures vs. 91 conventional mitral surgical repairs) shows a more complicated perioperative course for patients who underwent surgery which, however, is associated with significantly better results in terms of postoperative residual MR (0% for surgical repair vs. 11% in the Neochord group).

To have more reliable evidence, in 2016, in the United States, it started the ReChord clinical trial (NCT02803957) in which more than 500 patients affected by MR have been randomized between surgery and NeoChord. The study is currently not recruiting. It is important to highlight that it is also very difficult to compare a mature procedure with more than 40 years of practice behind with millions of patients treated overall versus a procedure that is currently performed only in selected centers with only 1500 cases performed. Moreover, it is also difficult to compare a procedure, in which the operator can use different techniques simultaneously on the leaflet (triangular‐quadrangular resection, chords, edge‐to‐edge, etc.) and on the annulus (closed, open, rigid, semirigid annuloplasty device) to increase leaflet coaptation to more than 5−7 mm, with respect to a procedure that could only reshape the leaflet by using long ePTFE chords.

The partially unsatisfying results, compared with surgery, cited above could also be linked to the inherent defect of all the transcatheter mitral devices actually available and represented by the possibility of treating only one component at a time of the mitral valve apparatus. This was also observed with the initial experience of edge‐to‐edge surgical repair without concomitant annuloplasty.^44^ The possibility of a combo approach, as successfully presented by Colli et al.^45^ (MitraClip and Amend device; Neochord and Amend device) and by Von Bardeleben et al.^46^ (Edwards Cardioband device + Neochord), is extremely encouraging but needs a more extensive evaluation. The same needs can be evocated for all the concomitant procedures described in the literature as Transapical TAVI and Neochord,^47^ off‐pump aortocoronary bypass and Neochord implantation ^48^ or for artificial chordal transapical implantation as possible management in case of MR recurrence.^24^

To conclude, the transapical artificial chord implantation with Mitralstitch device, as presented in their paper by Wang et al., seems to be safe and effective, with results comparable with the reported experiences with other devices. The outcomes of transapical chords implantations will surely benefit from the progressive diffusion of the procedure, bringing to a better knowledge of the device and to a further optimization in patients’ selection. The possibility of a future shift from the transapical to transfemoral approach is not prohibitive, as recently announced with first successful cases performed. What is really required is a significant evolution of the surgeon's education to embrace the concept of an ongoing transcatheter surgical procedure re‐evolution.

Matteo Saccocci have nothing to disclose. Andrea Colli, is a proctor for Neochord Inc., received consulting fee from Valgene Inc., and is a consultant for CoreMedic GmbH, received consulting fee from Techwald.
